# Development and Assessment of a Decentralization Framework in Iran Pharmaceutical Regulatory Systems Based on Good Governance Indicators

**DOI:** 10.5812/ijpr-165508

**Published:** 2025-09-30

**Authors:** Sajjad Esmaeili, Mohammad Peikanpour, Farzad Peiravian

**Affiliations:** 1 Department of Pharmacoeconomics and Pharma Management, School of Pharmacy, Shahid Beheshti University of Medical Sciences, Tehran, Iran

**Keywords:** Decentralization, Pharmaceutical Regulation, Good Governance, Iranian National Health System

## Abstract

**Background:**

Decentralization policies are essential for reforming administrative structures and improving service efficiency in government. Proper implementation of these policies promotes effective governance within administrative systems.

**Objectives:**

This study proposes a framework for decentralizing regulatory tasks in the pharmaceutical sector to improve good governance indicators.

**Methods:**

In the qualitative phase, an Interview Question Guide (IQG) was used to examine the decentralization framework through focused group discussions (FGDs) with relevant experts. The quantitative phase evaluated a sample of domestically produced and imported medicines, collecting perspectives from pharmaceutical companies, Iran Food and Drug Administration (IFDA) employees, and vice-chancelleries of food and drug affairs (VCFDA) staff at universities of medical sciences. The assessed indicators included transparency, rule of law, accountability and responsibility, effectiveness and efficiency, and stakeholder participation.

**Results:**

Experts agreed on delegating tasks related to scientific examination and drug procurement policy-making. For supervisory and inspection tasks, deconcentration with VCFDA oversight was recommended. Key suggestions included legislative amendments, strengthening regulatory infrastructure, reforming processes, and learning from past decentralization efforts. In the quantitative phase, 169 companies responded, with over 59% of respondents holding professional doctoral degrees. Among VCFDA respondents, 33 individuals participated, with more than 60% having over ten years of work experience. Transparency scored highest among pharmaceutical companies, while IFDA and VCFDA employees emphasized responsiveness and accountability. Statistical analysis revealed significant differences across all dimensions between IFDA and pharmaceutical company perspectives.

**Conclusions:**

Decentralization policies can improve good governance indicators in the pharmaceutical sector if critical requirements are met. Strengthening regulatory infrastructure and clearly defining tasks will enable effective use of governmental and non-governmental capacities.

## 1. Background

Evaluating governmental policy-making organizations is essential for evidence-based policy development and effective planning in any nation (1). A primary approach to improving service delivery involves reforming administrative bureaucracies, particularly through decentralization policies that enhance the speed and quality of governance (2). Since World War II, decentralization has emerged as a management strategy, transferring authority to lower levels to improve citizen engagement, accountability, and responsiveness (3). Structural reforms in healthcare systems have gained importance due to their extensive global impact. Decentralization in healthcare can boost efficiency (4), provided appropriate decentralization levels are defined and monitored effectively (5). From 1972 to 2019, approximately half of public health services in 75 countries were decentralized, with local governments playing a significant role during the COVID-19 pandemic (6). The success of decentralization depends not only on its structural design but also on its ability to promote democracy and good governance in local sectors (7). Decentralization programs, as outlined by Faguet, aim to improve governance performance, responsiveness, service efficiency, equitable access, and resource distribution (8). The International Monetary Fund (IMF) notes that decentralization, when supported by strong governance infrastructure, leads to better social outcomes (9). However, inadequate infrastructure can hinder good governance, causing issues such as insufficient skilled personnel, low stakeholder involvement, and weak service delivery (10).

The pharmaceutical sector is a vital focus for policymakers worldwide (11). Its complexity and vulnerabilities require a robust policy framework to ensure public access (12). In the Islamic Republic of Iran, the Iran Food and Drug Administration (IFDA) oversees policymaking for the supply and procurement of medical products, serving as the primary regulator of product quality and guideline development. The General Department of Medicines and Controlled Substances (GDMCS) manages administrative and technical affairs in the pharmaceutical sector, while the vice-chancelleries of food and drug affairs (VCFDA), located within universities of medical sciences, acts as local governance bodies in provinces, supporting IFDA’s administrative and supervisory roles. Systematic evaluation of these activities can track progress toward pharmaceutical system goals, identify gaps, and support comprehensive policy assessment (13). Integrating decentralization with good governance principles can enable phased decision-making and policy development.

## 2. Objectives

This study addresses how to implement a decentralized regulatory system in the pharmaceutical sector to achieve good governance based on performance standards. It contributes by proposing a framework for decentralization policies in the pharmaceutical sector, incorporating expert insights on decentralization levels, service processes, and relevant centers. Additionally, it establishes an evaluation framework for decentralized tasks based on good governance indicators.

## 3. Methods

This study was conducted in two phases: Qualitative and quantitative. The qualitative phase focused on developing a framework for decentralizing regulatory services in the pharmaceutical sector and creating a performance assessment tool to evaluate decentralization based on good governance indicators.

### 3.1. Development of Research Tools

An extensive literature review was conducted to inform the development of research tools, covering decentralization concepts and levels, good governance in healthcare and pharmaceutical systems, and governmental responsibilities in the public and private sectors under Islamic Republic of Iran laws. The review also included studies on the decentralization of governmental duties in healthcare and pharmaceutical systems and its impact on good governance indicators. For the qualitative phase, data collection tools were developed by identifying 38 regulatory services in the pharmaceutical sector, based on expert opinions and departmental input. After defining these services and their processes, Interview Question Guides (IQGs) were designed to specify service type, decentralization level, related processes, and target decentralization centers. The IQGs were tailored to four units within the GDMCS: Registration services (RS), inspection services (IS), pharmaceutical supply chain services (PSCS), and controlled substances services (CSS).

Decentralization levels were classified using Rondinelli’s framework, which includes delegation, deconcentration, devolution, and privatization (14). Delegation involves transferring defined tasks to non-governmental entities without transferring authority. Deconcentration transfers administrative responsibilities and authority to local governments (15). Devolution grants decision-making power to governmental or non-governmental entities with the necessary authority to perform tasks (16). In these levels, the central government retains ownership and decision-making authority. Privatization, defined as transferring ownership to non-governmental sectors, was considered equivalent to non-governmental service provision (17). These classifications guided the development of the IQGs. If experts deemed a service non-governmental, subsequent columns in the IQG were not completed. For governmental services, all relevant columns were filled out, as detailed in Table 1.

**Table 1.** Interview Question Guide Patterns for Assessing Decentralization Context in Pharmaceutical Regulatory Affairs

Row	Service Provided	Nature of Services	Level (Degree) of Decentralization	Processes that Can be Decentralized	Target Center for Decentralization
**1**	Insert any services	Governmental □	Delegation □	Insert processes related to any service	VCFDA □
		Non-governmental □	Deconcentration □		University and scientific centers □
			Devolution □		Other governmental organizations □
					Non-governmental associations □
					Private corporates □
					Other organizations □

To evaluate the effectiveness of decentralized services within the IFDA, the quantitative phase assessed a specific service: Sampling from the first batch of domestically produced and imported medicines. This inspection and supervision service, deconcentrated to the VCFDA, was chosen due to its high frequency of company requests, permit issuance, and inspection activities. Clear deconcentration guidelines also improved stakeholder data collection quality compared to other services. To create measurable indicators, five key dimensions were identified from the literature on good governance in the pharmaceutical sector and decentralization’s impact on governance indicators. The World Health Organization’s (WHO) Good Governance for Medicines (GGM) program defines a five-dimensional framework: Accountability, transparency, stakeholder participation, integrity and anti-corruption, and policy capacity development (18). Additional studies by Alphonse and Mekonnen highlight dimensions such as rule of law, transparency, accountability, effectiveness, and efficiency (19, 20).

A study tool was developed to measure five dimensions: Transparency, rule of law, accountability and responsibility, effectiveness and efficiency, and stakeholder participation. Indicators were customized based on literature and input from department heads overseeing service implementation. An initial questionnaire was created for a one-month period, targeting IFDA clients (pharmaceutical companies) and employees of IFDA and VCFDA. The questionnaire underwent face validity review by relevant experts to ensure accuracy and relevance. Reliability analysis, conducted for each group, showed acceptable Cronbach’s alpha results, detailed in Appendix 1 in Supplementary File.

### 3.2. Data Collection

In the qualitative phase, IQGs were developed, and focused group discussions (FGDs) were carefully planned with relevant experts. The FGDs involve participants with shared characteristics discussing a specific topic to share insights and experiences (21). These sessions reveal participants’ knowledge, attitudes, and motivations while capturing group dynamics (22). Purposive sampling selected experts based on IQG categories: (1) Current managers and experts from the GDMCS, (2) managers with relevant experience, and (3) pharmaceutical industry stakeholders linked to the department. Inclusion criteria required individuals in groups 1 and 2 to have over 5 years of regulatory sector experience and those in group 3 to have over 10 years in the pharmaceutical industry. Experts were grouped to ensure homogeneity and encourage knowledge sharing (23).

A total of 15 individuals were assigned to three groups for the RS IQG, 14 to three groups for the IS IQG, 13 to three groups for the PSCS IQG, and 7 to two groups for the CSS IQG. Sessions occurred between October and December 2022 following expert invitations. Textual data from FGDs were coded using MAXQDA software and analyzed with conventional qualitative methods. The FGD analysis involved thorough data extraction and concept integration (22, 24). Techniques such as grounded theory, content analysis, and discourse analysis were applied (25). Consistent questions, aligned with each IQG, were used to reach expert consensus on service types, decentralization levels, processes, and target centers. Content analysis categorized words and phrases in the text (26), while grounded theory supported the development of new concepts from expert opinions, identifying novel patterns not previously addressed in the literature.

In the quantitative phase, the statistical population was divided into two groups: Pharmaceutical companies and employees of the IFDA and VCFDA responsible for relevant services. For pharmaceutical companies, inclusion criteria included chief executive officers (CEOs), technical managers (TMs), and regulatory affairs managers (RAMs) with over 1 year of pharmaceutical industry experience. For IFDA and VCFDA employees, experts and managers with over 1 year of experience were selected. From 690 active pharmaceutical companies identified through the drug regulatory department, a sample size of 247 was calculated using Cochran’s formula. Questionnaires were distributed across company types (drug/APIs manufacturers, drug/APIs importers, medical device manufacturers/importers, and controlled materials producers) using stratified random sampling to ensure proportional representation of homogeneous groups (27).

For IFDA and VCFDA employees, the sample size matched the statistical population. Since only specific employees were involved in decentralization implementation, non-proportional quota sampling ensured representativeness when a specific sampling framework was unavailable (28). This method improved expert selection but introduced potential bias from self-reported responses, a noted study limitation. A total of 33 IFDA and 40 VCFDA employees were selected proportionally to the population, categorized by decentralized service units. This approach ensured the sample reflected the study population’s characteristics and provided insights into attitudes toward decentralization implementation within these organizations.

## 4. Results

### 4.1. Qualitative Phase

The FGDs were held separately for each service area after inviting relevant experts: The RS with 15 participants, IS with 14 participants, PSCS with 13 participants, and CSS with 7 participants. Table 2 outlines the characteristics of the FGD sessions and participants.

**Table 2.** Characteristics of the Focused Group Discussions Sessions and Participants

Types of Service; No. of Groups/Sessions	Session Duration (min)	Participants	Affiliation and Qualifications
**RS; 3 groups**			
1st group; 3 sessions	293	6	All PharmD (3 directors in GDMCS, others field experts)
2nd group; 3 sessions	264	5	Two Ph.D. (former GDMCS general directors), 1 Ph.D. (former GDMCS deputy), 1 Ph.D. (former GDMCS field director), 1 PharmD (former GDMCS general director)
3rd group; 2 sessions	196	4	All PharmD (active in pharmaceutical industry)
**IS; 3 groups**			
1st group; 2 sessions	212	6	All PharmD (4 directors in GDMCS, others field experts)
2nd group; 2 sessions	183	5	Two Ph.D. (former GDMCS general directors), 1 Ph.D. (former field deputy), 2 PharmD (former GDMCS general director and deputy)
3rd group; 1 session	85	3	All PharmD (active in pharmaceutical industry)
**PSCS; 3 groups**			
1st group; 2 sessions	224	5	All PharmD (2 directors in GDMCS, 2 field experts, 1 GDMCS deputy)
2nd group; 1 session	93	5	Two Ph.D. (former GDMCS general directors), 1 Ph.D. (former field deputy), 2 PharmD (former GDMCS general director and field director)
3rd group; 1 session	96	3	All PharmD (active in pharmaceutical industry)
**CSS; 2 groups**			
1st group; 1 session	101	4	One PharmD (GDMCS field director), 2 PharmD (field experts), 1 Master (field expert)
2nd group; 1 session	88	3	Two Ph.D. (former GDMCS general director and deputy), 1 PharmD (former GDMCS general director)

Abbreviations: RS, registration services; GDMCS, General Department of Medicines and Controlled Substances; IS, inspection services; PSCS, pharmaceutical supply chain services; CSS, controlled substances services.

**Figure 1.**
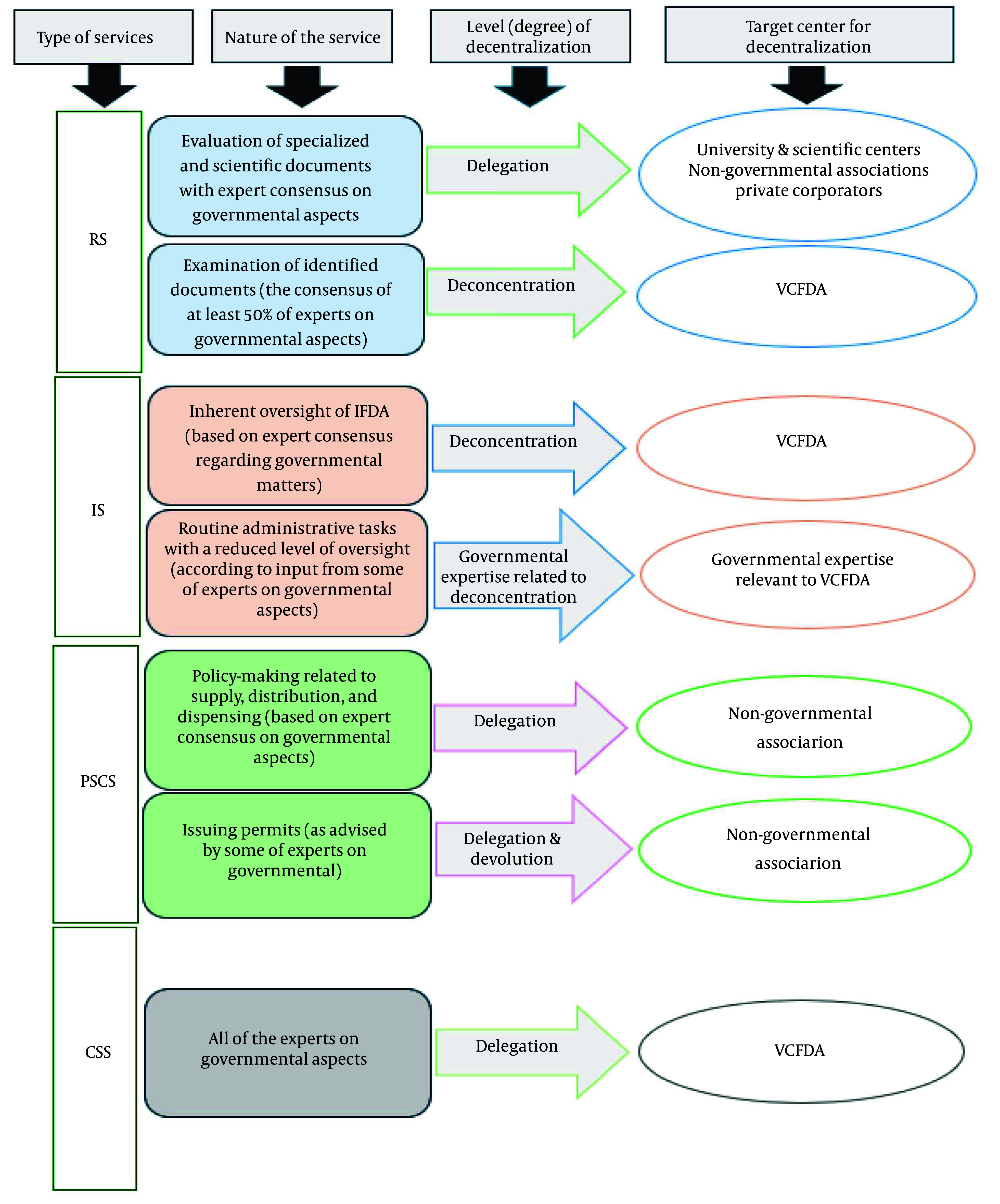
Summary of experts' consensus on decentralization framework for pharmaceutical regulatory services

Content analysis of the FGDs showed that the RS group reached a consensus to implement delegation for assessing specialized and scientific documents, while preferring deconcentration for other tasks based on expert feedback. The PSCS group supported relying on non-governmental associations, with delegation considered appropriate for achieving desired results. The IS and CSS groups emphasized using local government capacities (VCFDA). Summary results of the content analysis for each service are shown in Figure 1, with complete results available in Appendix 2 in Supplementary File.

Using grounded theory, textual data were coded collaboratively by two researchers, with discrepancies resolved through consultation with a third party. The analysis identified five main themes, 13 sub-themes, and 58 initial codes. The themes covered key aspects of decentralization policies within the Iran IFDA:

1. Developing and amending legal and regulatory frameworks in the IFDA.

2. Strengthening regulatory infrastructure to support decentralization policies.

3. Challenges in decentralizing IFDA functions to various sectors.

4. Reforming processes and structures to optimize decentralization policies.

5. Improving service efficiency and quality through decentralization.

Key sub-themes included topics such as IFDA decentralization guidelines, digitization of service delivery platforms, enhancement of administrative and technical infrastructure, inefficiencies in decentralizing tasks to local governments, resolution of organizational conflicts within the GDMCS, prioritization of decentralization by activity type, and improvement of regulatory affairs performance and agility. Detailed findings are available in Appendix 2 in Supplementary File, which provides a comprehensive overview of the grounded theory analysis.

### 4.2. Quantitative Phase

A questionnaire was distributed to 247 pharmaceutical companies, with 169 responding, yielding a response rate of 68.4%. Among these respondents, over 60% were female, 76.5% were aged 35 - 55, 59% held professional doctoral degrees, and 69.8% were TM within their companies. For the VCFDA, 33 of 40 distributed questionnaires were returned, achieving an 82.5% response rate. Over 60% of VCFDA respondents had more than ten years of work experience. The analysis calculated the median and standard deviation (SD) for each measured dimension. Among decentralized services, transparency had the highest median score, while the rule of law had the lowest, based on pharmaceutical company responses. Conversely, IFDA and VCFDA employees rated responsiveness and accountability highest, with stakeholder participation receiving the lowest median score. Complete results are presented in Table 3. 

**Table 3.** Descriptive Results of the Main Dimensions

Variables	Median	SD
**Pharmaceutical companies**		
Transparency	2.17	0.426
Rule of law	1.89	0.526
Accountability and responsibility	2.00	0.580
Effectiveness and efficacy	1.94	0.458
Stakeholder participation	2.00	0.518
**Employees of the IFDA and VCFDA**		
Transparency	2.60	0.417
Rule of law	2.42	0.504
Accountability and responsibility	2.90	0.318
Effectiveness and efficacy	2.54	0.492
Stakeholder participation	2.20	0.467

Abbreviations: SD, standard deviation; IFDA, Iran Food and Drug Administration; VCFDA, vice-chancelleries of food and drug affairs.

Analytical tests were conducted across three groups — employees of the IFDA, VCFDA, and pharmaceutical companies — using Kruskal-Wallis and Mann-Whitney U tests to evaluate differences in attitudes toward five key dimensions. A Kruskal-Wallis test was first applied to assess variations among all three groups across these dimensions. After detecting significant differences in all dimensions, pairwise comparisons were performed using the Mann-Whitney U test, with Bonferroni correction applied. Results showed significant differences between IFDA and VCFDA employees in all dimensions except responsibility and accountability. Similarly, significant differences were found across all dimensions between IFDA employees and pharmaceutical companies (Table 4). 

**Table 4.** Mann-Whitney Results of the Main Dimensions in the Three Groups

Variables	P-Value	Mean Rank
**The results between the VCFDA and the IFDA**		
Transparency	< 0.001	The VCFDA: 47.35
		The IFDA: 19.65
Rule of law	< 0.001	The VCFDA: 41.65
		The IFDA: 19.95
Accountability and responsibility	0.053	The VCFDA:34.33
		The IFDA: 28.28
Effectiveness and efficacy	< 0.001	The VCFDA: 44.88
		The IFDA: 22.12
Stakeholder participation	0.004	The VCFDA: 30.48
		The IFDA: 20.54
**The results between the VCFDA and pharmaceutical companies**		
Transparency	< 0.001	The VCFDA: 173.33
		Pharmaceutical companies: 87.47
Rule of law	< 0.001	The VCFDA: 167.92
		Pharmaceutical companies: 88.53
Accountability and responsibility	< 0.001	The VCFDA: 165.48
		Pharmaceutical companies: 89.01
Effectiveness and efficacy	< 0.001	The VCFDA: 173.27
		Pharmaceutical companies: 87.49
Stakeholder participation	< 0.001	The VCFDA: 136.32
		Pharmaceutical companies: 78.80
**The results between the IFDA and pharmaceutical companies**		
Transparency	0.016	The IFDA: 119.97
		Pharmaceutical companies: 97.89
Rule of law	0.002	The IFDA: 126.90
		Pharmaceutical companies: 94.80
Accountability and responsibility	< 0.001	The IFDA: 152.17
		Pharmaceutical companies: 90.46
Effectiveness and efficacy	0.004	The IFDA: 124.89
		Pharmaceutical companies: 96.93
Stakeholder participation	0.001	The IFDA: 117.57
		Pharmaceutical companies: 80.48

Abbreviations: VCFDA, vice-chancelleries of food and drug affairs; IFDA, Iran Food and Drug Administration.

## 5. Discussion

In this study, the decentralization framework within the IFDA, specifically focusing on the authority and responsibilities of the GDMCS, was examined through qualitative and quantitative phases. In the qualitative phase, FGDs with experts explored the decentralization framework, analyzing service types, decentralization levels, service-related processes, and targeted decentralization centers. Services were classified into four categories: The RS, IS, PSCS, and CSS. Participants included stakeholders with IFDA experience and members of non-governmental associations or syndicates.

In the quantitative phase, the effectiveness of decentralization policies was evaluated using good governance indicators: Transparency, rule of law, responsiveness and accountability, effectiveness and efficiency, and stakeholder participation. A key finding was the consensus among experts on the need to improve regulatory tools as a foundation for effective decentralization. They highlighted the importance of developing transparent algorithms for process decentralization, establishing a structured training framework for decentralized activities, and strengthening specialized human resources within the organization before implementing reforms. Continuous general and specialized training was also considered critical.

Furthermore, experts emphasized the need to develop and revise legal and regulatory frameworks within the IFDA. This includes updating laws related to decentralized tasks and creating legal mechanisms for service compensation to provide a clear structure for reform policies.

Implementing these recommendations — such as enhancing infrastructure, refining legal frameworks, and coordinating efforts among relevant departments — is expected to support effective decentralization within the IFDA. These steps will clarify governance responsibilities and establish transparent decentralization patterns, ultimately strengthening the IFDA’s regulatory capacity as the central authority for policy execution. Najafikhah’s 2017 study underscored the importance of distinguishing between privatization and decentralization when determining private sector involvement in outsourcing programs, based on ownership, control requirements, and specific duties (29).

Another essential factor for successful decentralization is the development or amendment of relevant laws and regulations in the pharmaceutical sector. Specific regulatory measures have been designed to protect stakeholders’ confidential information, fostering trust and encouraging their participation in policymaking reforms. Financial considerations, including costs associated with various centers and their allocation, require legal pathways to establish appropriate tariffs for each activity within the financial decentralization framework. Several studies highlight the critical role of governments in enacting laws to support effective decentralization, identifying it as a key indicator of good governance. Mohammed’s 2016 study identified the facilitation of governance-related laws as a core strategy for decentralization implementation (30).

Experts also stressed the importance of creating a clear policy framework for successful decentralization by reviewing past efforts and learning from their shortcomings to inform future policies. They noted inefficiencies in decentralization at the local government level, particularly where the IFDA deconcentrated services to the VCFDA without ensuring adequate infrastructure, resulting in challenges being shifted between governance structures. Trimurni and Mansor’s 2020 study emphasized that administrative decentralization (deconcentration) is closely tied to improving technical capacities, including hardware and software skills, at the provincial level to build infrastructure for deconcentrating responsibilities and empowering local authorities to design health policies, plan, and innovate healthcare programs tailored to community needs (31).

Viphonephom et al. in 2024 noted that without a balanced distribution of power across governance levels and improvements in infrastructure — such as trained human resources and communication technologies — decentralization can lead to inequities and uneven structural development (32). Based on expert consensus, the next steps involve inter-agency negotiations to strengthen regulatory infrastructure and develop relevant regulations within the Iran IFDA. Prioritization is essential to determine decentralization levels and transferable centers, taking into account stakeholder confidentiality, service types, legal obligations, and the capacities of specialized scientific centers willing to provide services. These efforts aim to reinforce the central government’s role in guiding effective decentralization policies by optimizing infrastructural capabilities, ensuring reform success. Kyriacou’s 2015 study highlights that fiscal decentralization can reduce regional inequalities in countries with strong governance quality (33).

In the context of pharmaceutical regulatory decentralization, the IFDA, as the primary regulator, must develop a targeted plan to minimize implementation challenges and maximize decentralization benefits. Over 50% of experts emphasized benefits such as updating standards through decentralized administrative affairs in the private sector, focusing on long-term goals like standards improvement and export growth, and accelerating tasks through decentralization. Sumah and Baatiema’s 2018 study stresses the need for careful planning to anticipate decentralization requirements and outcomes, noting that effective decentralized policymaking can support centralized financial policies, fair wage setting, and balanced workforce distribution (34).

In the RS sector, experts underscored the importance of delegation and deconcentration for service decentralization. Given that RS processes involve sensitive documents and confidential information related to pharmaceutical company development, over 70% of experts opposed full devolution of authority. They supported decentralizing processes like license issuance to relevant departments, with final evaluation and issuance remaining under central government control. Concerns were raised about inadequate infrastructure in the VCFDA, including shortages of specialized human resources and limitations in hardware and software.

In the IS sector, experts agreed on an appropriate level of deconcentration for these government services. Given the critical supervisory role in IS, stakeholders emphasized VCFDA involvement at this decentralization level. Key recommendations included improving infrastructure and empowering VCFDA decision-making to deliver effective services. Experts also noted recent advancements in IFDA’s information systems and dashboards, which have enhanced information transfer between government departments. Jongudomsuk and Srisasalux’s 2012 study on Thailand’s health system decentralization highlights the importance of central government support in strengthening local government capacities to assume new responsibilities (35).

In the PSCS sector, experts stressed the importance of ensuring patient access to necessary medications. For policymaking in this sector, most experts supported delegation at appropriate levels but emphasized that PSCS activities, closely tied to drug procurement and provisioning, should remain under IFDA control to maintain centralized decision-making and planning. By enhancing data and information networks, the IFDA can effectively plan decentralization efforts, leveraging the capacities of relevant departments to boost competitiveness, safeguard stakeholder confidentiality, ensure a timely supply of quality drugs, and improve their distribution and dispensing.

Lima’s 2013 study suggests that a hybrid system can enhance drug procurement performance and responsiveness to patient needs by decentralizing administrative and financial tasks, such as planning, procurement, and budgeting, while maintaining centralized control over procurement, provisioning, and policymaking (12). Similarly, Chen et al.’s 2021 research indicates that managers of larger facilities and high-level centers have greater decision-making authority in providing essential drugs compared to those in smaller centers (36).

In the decentralization of sampling for both domestically produced and imported drugs, a deconcentration approach was applied. Feedback from pharmaceutical companies revealed that transparency scored above average, while the rule of law scored below average. Companies raised concerns about inadequate anti-corruption measures, insufficient mechanisms for handling stakeholder objections, and inconsistent enforcement of laws and regulations by the IFDA and the VCFDA. Positive aspects included employee commitment and confidentiality within governmental organizations, as well as written result notifications, which bolstered the Transparency Index. However, issues persisted with the irregular issuance and updating of circulars, and stakeholders noted that IFDA guidelines lacked the quality and accessibility needed for effective supervision.

Regarding efficiency and effectiveness, pharmaceutical companies expressed dissatisfaction with the limited reduction in IFDA visits and improvements in review procedures following central government feedback. They emphasized the need for extensive training and experience in sampling and related tasks, which were often lacking in deconcentration processes outside the IFDA. This underscores the importance of robust support and capacity-building for local entities to participate effectively in decentralized service delivery. Another study on healthcare decentralization in Portugal, Brazil, and Pakistan highlights the need to strengthen implementation strategies, improve equity in service access, and develop robust measurement and monitoring mechanisms (37).

Responses from IFDA and VCFDA employees showed that responsiveness and accountability scored highest (2.90), while stakeholder participation scored lowest (2.20). Analytical tests identified significant differences among pharmaceutical company representatives, IFDA employees, and VCFDA employees. Notably, IFDA employees reported lower satisfaction than VCFDA employees across all dimensions except responsiveness and accountability. However, a limitation of the study is the potential for bias, as respondents in both groups reported on their own performance.

In summary, decentralization in the pharmaceutical regulatory sector has encountered challenges, resulting in suboptimal outcomes. These challenges include:

1. Unclear separation of duties and authorities: The decentralization process suffered from an ambiguous division of responsibilities and authorities among agencies involved in deconcentrated activities. This lack of clarity caused inefficiencies, wasted governmental time and resources, including human resources, and shifted problems from the central government to other sectors without addressing underlying infrastructure deficiencies.

2. Infrastructure and resource constraints: Responsibilities were transferred to local government entities without sufficient infrastructure improvements or capacity-building measures. Consequently, this led to inconsistent service quality and heightened uncertainties regarding responsiveness in service delivery.

3. Deficiencies in regulatory and legal frameworks: Decentralization to local governments proceeded without developing necessary regulations or amending existing laws. This absence of administrative safeguards hinders evidence-based policymaking and weakens governance structures intended to achieve good governance indicators.

Research by Jebessa emphasizes that decentralizing authority and resources can create governance challenges, requiring a clear separation of powers and responsibilities, along with effective oversight and balance among agencies (7). Similarly, Dagneh’s 2022 study in Ethiopia highlights that local governments often lack full decision-making authority, resulting in issues such as shortages of skilled personnel, uncommitted political leadership, low stakeholder participation, and inadequate service delivery (10).

### 5.1. Conclusions

Decentralization policies are critical for improving good governance indicators, provided key conditions are met. These include strengthening legal, regulatory, financial, and operational frameworks, promoting policymaking with robust stakeholder involvement, and prioritizing services and processes suitable for decentralization. Fulfilling these requirements can drive significant administrative transformation and enhance the efficiency of service delivery.

As a central government agency, the IFDA adopts a targeted decentralization approach guided by a specific framework. Given limitations in legal, financial, and human resources within the government sector, the IFDA collaborates with non-governmental entities, particularly for activities requiring scientific and specialized expertise. This strategic partnership leverages local government entities as regulatory mechanisms, preserving governmental oversight while delivering effective services to stakeholders in the health goods sector.

For services like procurement policy, distribution, and provision of medicines and medical equipment, which demand greater centralization, the IFDA prioritizes developing managerial dashboards, implementing process automation, and transparently updating relevant guidelines. These efforts aim to streamline policies and improve equitable access to quality health goods for the population. This approach reflects the IFDA’s commitment to strategic decentralization while ensuring efficient and effective governance in essential healthcare services.

ijpr-24-1-165508-s001.pdf

## Data Availability

The dataset presented in the study is available on request from the corresponding author during submission or after publication.
