# Supplementary material for: Development and Assessment of a Decentralization Framework in Iran Pharmaceutical Regulatory Systems Based on Good Governance Indicators
Source: Iran J Pharm Res. 2025 Sep 30;24(1):e165508. doi: 10.5812/ijpr-165508 (PMC12534734; doi:10.5812/ijpr-165508)
Supplement: ijpr-24-1-165508-s001.pdf [file ijpr-24-1-165508-s001.pdf]

Appendix 1: main indicators in the questionnaire of decentralization assessment- reliability analysis of the questionnaire

Abbreviations:

(IFDA): Iran Food and Drug Administration

(VCFDA): Vice-Chancelleries of Food and Drug Affairs in Universities of Medical Affairs

(NGAs): Non- Governmental Associations

Appendix 2: content and grounded theory analysis results of qualitative phase

Table S1.1. Assessment indicators in decentralization of the First Batch of Domestically Produced and Imported Medicines (FBDPIM) based on good governance perspective

Questions(indicators)	Responses
**Transparency**	
Regular and up to date publication of instructions by the IFDA	at all ☐ to some extent ☐ quite ☐ no idea ☐
Confidentiality of people related to documents review in the VCFDA	at all ☐ to some extent ☐ quite ☐ no idea ☐
Confidentiality of people related to documents review in the IFDA	at all ☐ to some extent ☐ quite ☐ no idea ☐
Transparency in method of document review in the VCFDA	at all ☐ to some extent ☐ quite ☐ no idea ☐
Transparency in decision process of the IFDA's managers and experts	at all ☐ to some extent ☐ quite ☐ no idea ☐
Systems related to the FBDPIM cause promotion of transparency	at all ☐ to some extent ☐ quite ☐ no idea ☐
Declaration of the results of documents review written	at all ☐ to some extent ☐ quite ☐ no idea ☐
**Rule of law**	
Announcing of regular instruction (law) for deconcentration the FBDPIM	at all ☐ to some extent ☐ quite ☐ no idea ☐
Fair application of the related instruction (law) for all of clients in the VCFDA	at all ☐ to some extent ☐ quite ☐ no idea ☐
Fair application of the related instruction (law) for all of clients in the IFDA	at all ☐ to some extent ☐ quite ☐ no idea ☐
Effectiveness and efficacy of the related instruction (law)	at all ☐ to some extent ☐ quite ☐ no idea ☐
lack of ambiguity in the related instruction (law)	at all ☐ to some extent ☐ quite ☐ no idea ☐

Requirements related to confidentiality of information in the related instruction (law)	at all ☐	to some extent ☐	quite ☐	no idea ☐
Consideration of stakeholder objection in the related instruction (law)	at all ☐	to some extent ☐	quite ☐	no idea ☐
Development of prevention of corruption in the related instruction (law)	at all ☐	to some extent ☐	quite ☐	no idea ☐
Development of the related instruction (law) evidence based	at all ☐	to some extent ☐	quite ☐	no idea ☐
**Accountability and Responsibility**				
Timely responsiveness of stakeholder demands by the VCFDA	at all ☐	to some extent ☐	quite ☐	no idea ☐
Timely responsiveness of stakeholder demands by the IFDA	at all ☐	to some extent ☐	quite ☐	no idea ☐
Handling stakeholder objection by the IFDA about results of the FBDPIM	at all ☐	to some extent ☐	quite ☐	no idea ☐
Designing of process for stakeholder objection by the IFDA about results of the FBDPID	at all ☐	to some extent ☐	quite ☐	no idea ☐
Maintaining the human dignity of the client in the VCFDA	at all ☐	to some extent ☐	quite ☐	no idea ☐
Maintaining the human dignity of the client in the IFDA	at all ☐	to some extent ☐	quite ☐	no idea ☐
**Effectiveness and Efficacy**				
Duration of the FBDPIM is in the announcement period	at all ☐	to some extent ☐	quite ☐	no idea ☐
Improvement of document review after giving feedback to the VCFDA by the IFDA	at all ☐	to some extent ☐	quite ☐	no idea ☐
Adequacy of trainings from central government	at all ☐	to some extent ☐	quite ☐	no idea ☐
Reduction of referral to the IFDA after deconcentration the FBDPIM	at all ☐	to some extent ☐	quite ☐	no idea ☐
Timely and regular announcement of results related to the FBDPIM to the companies by the IFDA	at all ☐	to some extent ☐	quite ☐	no idea ☐
Increasing the efficiency of decentralization by the relevant systems	at all ☐	to some extent ☐	quite ☐	no idea ☐
Correct implementation of prevention of corruption in the VCFDA	at all ☐	to some extent ☐	quite ☐	no idea ☐
Correct implementation of prevention of corruption in the IFDA	at all ☐	to some extent ☐	quite ☐	no idea ☐
Improvement of confidentiality of information related to the stakeholders in the VCFDA	at all ☐	to some extent ☐	quite ☐	no idea ☐
Considering confidentiality of information during implementation of the FBDPIM in location of compa	at all ☐	to some extent ☐	quite ☐	no idea ☐
Improvement of confidentiality of information related to the stakeholders in the IFDA	at all ☐	to some extent ☐	quite ☐	no idea ☐
Monitoring decentralization of the FBDPIM by the IFDA at certain intervals	at all ☐	to some extent ☐	quite ☐	no idea ☐
Implementation of the instruction (law) completely by the VCFDA	at all ☐	to some extent ☐	quite ☐	no idea ☐
Sending the samples to the IFDA with adherence to proper storage protocols	at all ☐	to some extent ☐	quite ☐	no idea ☐
Implementation of quality of the FBDPIM based on the IFDA's instruction	at all ☐	to some extent ☐	quite ☐	no idea ☐
Controlling the forms related to the FBDPIM by the VCFDA When referring to the company	at all ☐	to some extent ☐	quite ☐	no idea ☐
**Stakeholder participation**				
Utilizing the opinions of the NGAs representative in formulation/amendment of relevant instruction	at all ☐	to some extent ☐	quite ☐	no idea ☐
Informing the NGAs representative about the action and decision of the IFDA	at all ☐	to some extent ☐	quite ☐	no idea ☐
Informing the NGAs representative about the action and decision of the VCFDA	at all ☐	to some extent ☐	quite ☐	no idea ☐
Adequate informing the stakeholders about publication of information relevant the FBDPIM	at all ☐	to some extent ☐	quite ☐	no idea ☐
Improvement of the document review with feedback from stakeholder to the VCFDA	at all ☐	to some extent ☐	quite ☐	no idea ☐
Improvement of the document review with feedback from stakeholder to the IFDA	at all ☐	to some extent ☐	quite ☐	no idea ☐

Table S1.2. results of reliability analysis

dimensions	Cronbach’s alpha (for respondents of pharmaceutical company)	Cronbach’s alpha (for employees of both IFDA and VCFDA)
Transparency	0.868	0.705
Rule of law	0.916	0.895
Accountability and Responsibility	0.926	0.621
Effectiveness and Efficacy	0.948	0.971
Stakeholder participation	0.930	0.961

Table S2.1. Content analysis results of RS IQG

Type of Services	Nature of Services	Level (degree) of Decentralization	Processes that can be Decentralized	Target Center for Decentralization
Issuing the license for domestically produced medicine	governmental	delegation	>80% of the experts<br><br>(reviews of documents and Common Technical Document (CTD))	The expert consensus (Non- governmental associations (NGAs), University & Scientific Centers (USCs) and private corporates)
Issuing the license for imported medicine	governmental	50% of the experts on delegation, 50% of the experts on deconcentration	>90% of the experts<br><br>(Good Manufacturing Practice (GMP) certification in country of origin, reviews of documents and Common Technical Document (CTD))	> 50% of the experts<br><br>(NGAs and VCFDA)
Issuing/extending license of the technical manager	governmental	deconcentration	The expert consensus (documents review and inquiry of the fields)	The VCFDA
Issuance of in-principle approval of medical institutions	70% of experts (governmental), 30% of experts (non-governmental)	>70% of experts (delegation & deconcentration)	Documents review	Consensus of experts (the VCFDA, NGAs and private corporate)
Issuing the license for foundation of medical institutions	70% of experts (governmental), 30% of experts (non-governmental)	33.3% of experts(delegation), 33.3% of experts(deconcentration),	The expert consensus (site visit and document review)	>70% of experts (the VCFDA and NGAs)

		33.3% of experts(devolution)		
Activity licensing of medical institutions	governmental	Expert consensus (deconcentration & devolution)	Document review & issuance of approval	The VCFDA
Issuing/extending license of biologic products	governmental	delegation	Expert consensus (CTD review and Pre-registration inspection)	NGAs and USCs
Issuing the license for the Active Pharmaceutical Ingredients (APIs) domestically produced	governmental	Expert consensus (delegation & deconcentration)	in-principle approval, inspection and documents review	USCs and the VCFDA
Issuing the license for imported APIs	55% of experts(governmental), 45% of experts(non-governmental)	60% of experts related to governmental (devolution)	>80% of experts related to governmental (source registration and sampling)	>60% of experts (USCs, NGAs and the VCFDA)
extending license of APIs domestically produced/imported	70% of experts (governmental), 30% of experts (non-governmental)	>60% of experts (delegation)	Experts consensus (Documents review and sampling)	NGAs and the VCFDA

Table S2.2. Content analysis results of IS IQG

Type of Services	Nature of Services	Level (degree) of Decentralization	Processes that can be Decentralized	Target Center for Decentralization
Issuing the Good Manufacturing Practice (GMP) certification	governmental	70% of experts (delegation), 30% of them (devolution)	Experts consensus<br><br>(visit the location, get the Corrective Actions and Preventive Actions (CAPA))	The expert consensus (private corporates and NGAs)
Dealing with the Recall	governmental	60% of the experts (deconcentration)	Experts consensus<br><br>(get the report, sampling and Announcing response to the company)	The VCFDA
Dealing with the complaint	governmental	50% of the experts (deconcentration)	The expert consensus (on-site inspection, sampling and compilation of the final report)	The VCFDA
Verification of construction site	55% of experts (governmental), 45% of experts (non-governmental)	>60% of experts related to governmental (deconcentration)	>80% of experts (on-site inspection and compilation of the final report)	The VCFDA
The sampling	55% of experts (governmental), 45% of experts (non-governmental)	50% of experts related to governmental (deconcentration)	The expert consensus (sampling in the location, uploading the information in the system)	The VCFDA

Visiting the pre-verification of APIs manufacturer	50% of experts (governmental), 50% of experts (non-governmental)	The experts consensus related to governmental (deconcentration)	The expert consensus (on-site inspection and reporting)	The VCFDA
Post Marketing Quality Control (PMQC)	governmental	delegation	Expert consensus (sampling and Sending to the laboratory)	The expert consensus (The VCFDA and USCs)
Supervision of product destruction	>80% of experts (non-governmental)	-	-	-
Releasing the travel medicines from customs	>70% of experts (non-governmental)	-	-	-
Investigating the map of manufacturing factories	Non-governmental	-	-	-

Table S2.3. Content analysis results of PSCS IQG

Type of Services	Nature of Services	Level (degree) of Decentralization	Processes that can be Decentralized	Target Center for Decentralization
Policy-making and issuing the medicines with temporary license	governmental	50% of experts (delegation)	Holding the tender and uploading the results in system	50% of experts (NGAs)
Monitoring the pharmaceutical supply chain	56% of experts (governmental), 44% of experts (non-governmental)	60% of the experts related to governmental (delegation)	Experts consensus<br><br>(monitoring the information in rolling forecast system)	NGAs and private corporate
Pricing the medicines	governmental	delegation	investigation of pricing forms and initial calculation of the prices	NGAs and USCs
Supervision of medicine distribution	>75% of experts (governmental)	>70% of experts (deconcentration & delegation)	Experts consensus (supervision on technical managers, prices and fair distribution of medicines)	(The VCFDA, NGAs and private corporate)
Issuing the export license	55% of experts (governmental), 45% of experts (non-governmental)	60% of experts (delegation), 40% of them (devolution)	The expert consensus (Inquiring from the relevant field and preparing a draft)	NGAs

Allocation of currency	governmental	60% of experts (delegation)	The expert consensus (Summarizing and presenting the list to the committee)	NGAs
Planning and policy-making the imported medicines that are registered	governmental	60% of experts (delegation)	Expert consensus (investigating the CPT price and history of imported medicines)	>50% of experts (NGAs and private corporate)
Policy-making the APIs	governmental	Delegation	Expert consensus (policy of commercial profit and import condition)	NGAs
Issuing the license of medicine release	>60% of experts (non-governmental)	-	-	-

Table S2.4. Content analysis results of CSS IQG

Table S2.5. Grounded Theory results of focus group discussions

themes	Sub-themes	Primary codes

Type of Services	Nature of Services	Level (degree) of Decentralization	Processes that can be Decentralized	Target Center for Decentralization
Issuing the international licenses of the controlled materials and medicines	governmental	delegation	Investigation of proforma and other relevant documents	The VCFDA
Policy-making the controlled materials and medicines domestically produced	governmental	delegation	Experts consensus<br><br>(preparation of information bank about production and consumption of materials)	The VCFDA
Supervision of weighing, rationing and delivery of opioids	governmental	delegation	Experts consensus (On-site presence and reporting)	The VCFDA
Remittance of controlled materials and medicines	governmental	delegation	Experts consensus (documents review)	The VCFDA
Providing annual reports of production and consumption of controlled materials and medicines to international authorities	governmental	delegation	The expert consensus (preparation of information bank completely)	>50% of experts (the VCFDA and NGAs)

Developing and amending the necessary legal and regulatory frameworks in the IFDA	Set of instructions related to decentralization by IFDA	Development of requirements and standards by the government; development of relevant instructions based on stakeholders' comments; considering an appeal process for stakeholders; the necessity for the government to have a long-term vision for policy-making; considering confidentiality commitments from companies for decentralized processes; obliging the related corporates to accept responsibility in the Memorandum Of Understanding (MOU)
	Development of service compensation mechanisms by the IFDA	Considering the costs of decentralization by the IFDA; taking financial commitments into account; clarifying the tariffs for decentralized activities; considering the process for receiving service fees from pharmaceutical companies
Strengthening regulatory infrastructures for the implementation of decentralization policies	Serious attention to the creation of monitoring and supervision infrastructures	Random inspections of the IFDA from services provided by the related centers; providing the specifications of personnel related to private corporates to the IFDA; the necessity of periodic monitoring of decentralized activities; designing specialized checklists related to the decentralization of each process for monitoring; implementation of decentralization on a three to six month basis with subsequent monitoring
	Intelligentization of service delivery in system platforms	Designing specific dashboards for decentralization through Tracking and Tracing and Authentication Control (TTAC) system; digitizing some processes before implementing decentralization; enhancing the automation of highly sensitive services such as Recall without implementation of decentralization; creating coordination between the approval of the technical manager and the relevant department in issuing permission of distribution within the TTAC system; developing shortage and identification dashboards within the TTAC system to reduce the workload of the IFDA

	Strengthening administrative and technical infrastructures	Clarifying the algorithm for decentralization of processes; developing a training framework by the IFDA for decentralized activities; the necessity of strengthening specialized human resources within the IFDA before decentralization; providing both general and specialized training continuously; the necessity of ongoing interactions between decentralized centers and the IFDA; conducting territorial arrangement about manufacturing factories and aligning them with planning; necessity of documentation as key prerequisites for monitoring decentralization; defining the timeline for implementing the decentralized process
Issues in the decentralization of IFDA to various sectors	Challenges related to the IFDA's commitment in international conventions	Issues related to international conventions such as PIC/S in implementation of decentralization; the impossibility of decentralization related to Good Manufacturing Practice (GMP); serious problems in export activities in case of decentralization of GMP to a corporate other than the IFDA
	Inefficiency in decentralization of activities to the local government	Lack of willingness among professors of university to take responsibility for decentralization; serious legal challenges for decentralization to academic centers; increasing the IFDA workload with decentralization to the VCFDA; insufficient diversity of specialists in the VCFDA compared to the IFDA
	Problems related to the tasks of the IFDA in decentralization	Confidentiality and responsibility of governmental affairs as major concerns in decentralization; increasing problems in case of decentralization without infrastructure development; the necessity of assessing the challenges related to implementation of decentralization in past years
Process and structure reform; the necessity of implementing	Resolution of organizational conflicts in GDMCS and facilitation of processes	Reviewing processes from initial to final to gain necessary oversight; creating uniform procedures for common processes in different departments; resolving conflicts in departmental processes before

decentralization policies optimally		decentralization; properly separating between departments tasks to prevent conflicts
	Developing a clear framework of the structures and activities of the GDMCS	The necessity of transparent presence of the private sector in the process of decentralization; administrative processes related to drug procurement and supply sector as non-governmental affairs; changing the IFDA's role to the recipient of copies in the deconcentration of process to the VCFDA; issuing the final license and vote of the commission the as governance affairs; creating decision-making power in the VCFDA for optimal management of decentralized activities
	Prioritization of decentralization according to the type of activity	Determining the level of decentralization through collaboration with experts; deconcentration of services to the VCFDA in services with high confidentiality; decentralization of document review processes to academic centers due to relative governance trust; considering specialized infrastructure requirements in decentralization of process to various corporates; the necessity of utilizing the scientific capabilities in faculties of pharmacy to advancing relevant affairs
Enhancing efficiency and quality of services through decentralization policy implementation	Improving the performance and agility of regulatory affairs	Developing and updating standards through decentralization of executive tasks to the private sector; increasing resilience and reducing system vulnerability in decentralization; developing and updating guidelines by the IFDA after implementation of decentralization
	Development of relevant infrastructures	The IFDA focus on long-term goals such as promoting standards and increasing exports; economic development and production growth through implementation of decentralization; accelerating operations through decentralization
